# 399. Reducing Hospital-Onset C. diff with a Provider-Driven Emergency Department Ordering Algorithm

**DOI:** 10.1093/ofid/ofac492.477

**Published:** 2022-12-15

**Authors:** Jill Holdsworth, Aaron Milloy, Jordan Kaylor, Rita Richardson, Patrick Meloy

**Affiliations:** Emory University Hospital Midtown, Atlanta, Georgia; Emory University Hospital Midtown, Atlanta, Georgia; Emory University School of Medicine, Atlanta, Georgia; Emory University Hospital Midtown, Atlanta, Georgia; Emory University School of Medicine, Atlanta, Georgia

## Abstract

**Background:**

*Clostridoides difficile* (C. diff) can cause diarrhea and inflammation of the colon. It is one of the most common causes of heathcare-associated infections (HAIs), estimated to cause almost half a million illnesses and thousands of deaths in the United States each year. Studies have estimated that this HAI costs up to $4.8 billion each year in excess healthcare costs for acute care facilities. Early identification, treatment and initiation of isolation precautions is crucial to patient care and transmission reduction, making the Emergency Department ideal partners in efforts to reduce C. diff infections.

**Methods:**

A C. diff testing algorithm was introduced specific to the Emergency Department in March, 2021, promoting earlier testing and isolation of patients suspected of having C. diff. Emergency medicine clinicians were awarded the "Golden Spore" Award weekly when appropriately initiating the C. diff testing and isolation, provided the patients met criteria, or were clinically suspected of having a C. diff infection.

C. diff ordering algorithm

**Figure:**
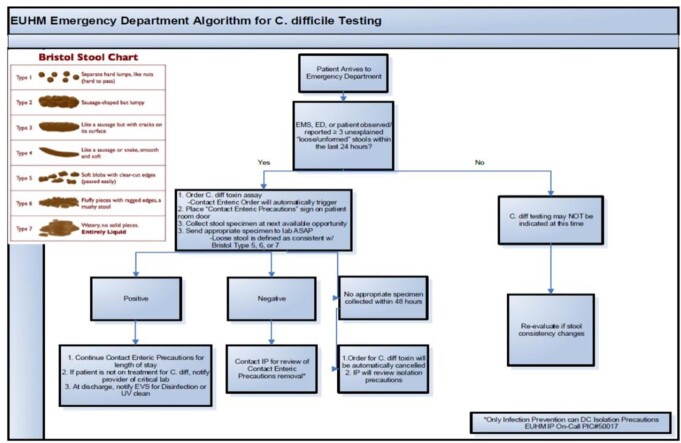

Ordering algorithms were printed and placed in the clinician's work area to facilitate increased ordering of isolation and testing of potentially infected patients.

GoldenSporeAward

**Figure:**
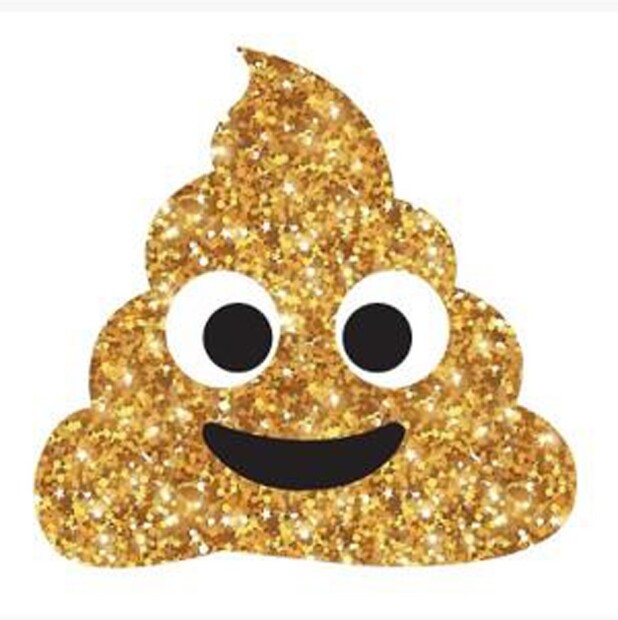

The Golden Spore is awarded weekly to emergency clinicians who successfully identify and isolate C. diff patients prior to admission.

**Results:**

As a result of the COVID-19 pandemic, there were varying degrees of challenges for implementation of the program. Emergency clinicians sent 199 C. diff tests pre-intervention (3/15/20-3/14/21) and 234 tests post-intervention (3/15/21-3/14/22). Pre-intervention, 44 patients were found to be C. diff positive prior to admission, compared to 62 post-intervention. Clinicians enjoyed the "golden spore" award and were excited to participate.

**Conclusion:**

The program is ongoing, though the initial results are encouraging. There was an immediate increase in the testing and subsequent isolation of patients while in the ED. Infection prevention and emergency medicine clinicians worked together to boost their numbers, and the "golden spore" continues to be awarded in weekly newsletters. Clinicians indicated they enjoyed the engagement, and found that the small change in a routine process improved their baseline awareness of potential C. diff patients, leading to an increase in the number of tests and subsequent discovery of C. diff positive patients. C. diff champions were also named and the algorithm will be shared with additional emergency departments within our system to continue to improve detection and prevention of hospital associated infections.

**Disclosures:**

**All Authors**: No reported disclosures.

